# Pediatric patient with linearly distributed bullae

**DOI:** 10.1016/j.jdcr.2023.06.048

**Published:** 2023-07-17

**Authors:** Alanoud Alhadyani, Ansley DeVore, Emily Gaster, Dirk Elston

**Affiliations:** a Department of Dermatology, Medical University of South Carolina, Charleston, South Carolina; b College of Medicine, Medical University of South Carolina, Charleston, South Carolina; c Department of Dermatopathology, Medical University of South Carolina, Charleston, South Carolina

**Keywords:** bullosis diabeticorum, bullous diabeticorum, bullous eruption of diabetes mellitus, diabetic bullae

## History

**Fig 1:**
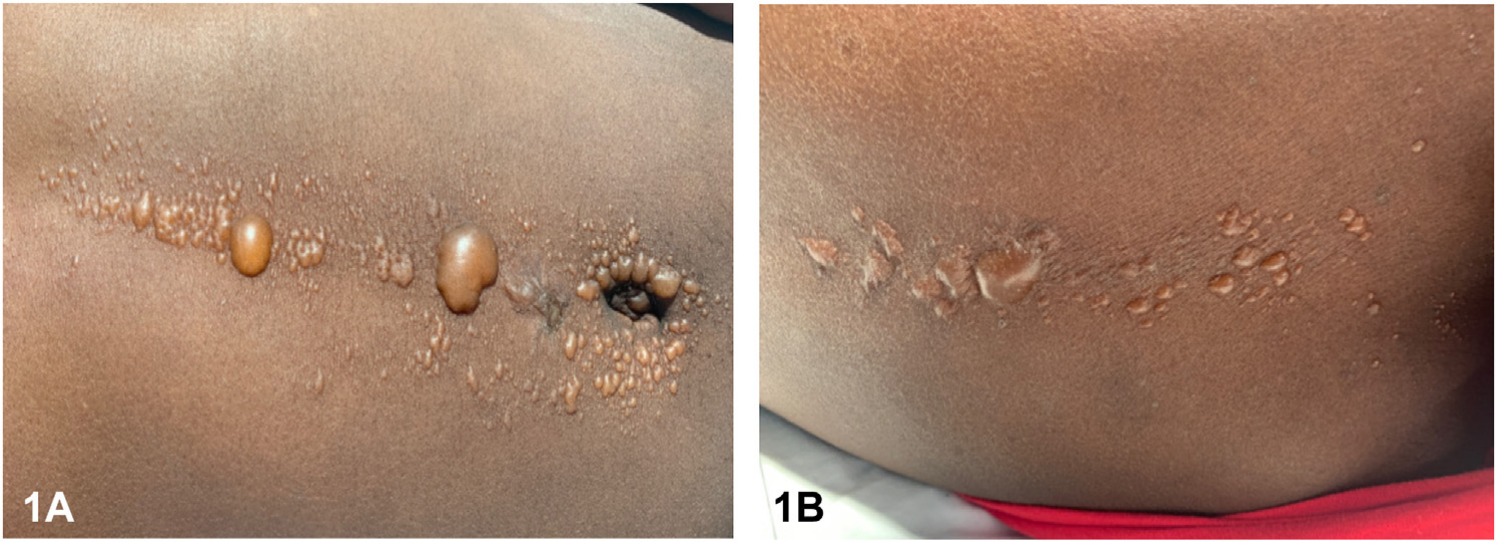
Tense bullae and vesicles on central abdomen, around the umbilicus, and on *mid-upper* back.

**Fig 2:**
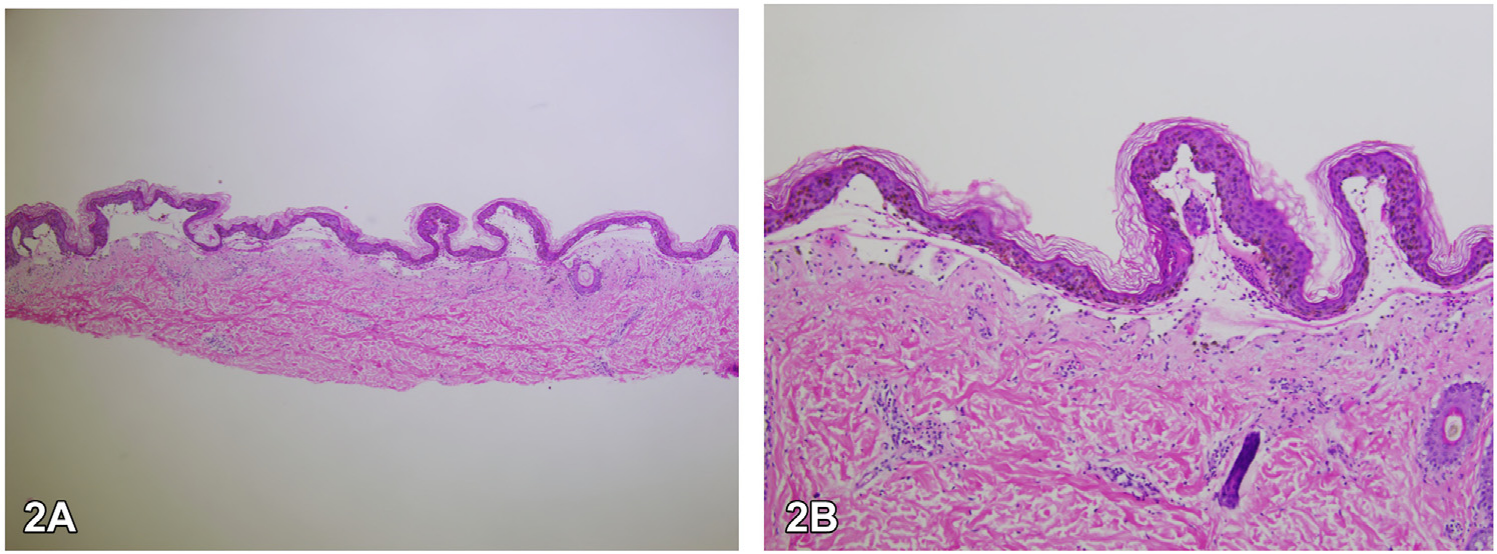
Hematoxylin and eosin stain (H&E) (4× and 10×) showing subepidermal separation with scattered neutrophils and necrotic keratinocytes.

A 17-year-old female with history of type 1 diabetes mellitus presented with nausea and emesis found to be in diabetic ketoacidosis. She had not been taking any medications prior to admission. HbA1c was 13.7% and glucose levels were over 500 mg. Fluids and insulin continuous infusion were started. On day 1 of admission, linear bullae and vesicles developed vertically along her abdomen and back (Fig 1). No external frictional forces were noted during exam or history. Negative for acral, mucosal, or ophthalmic involvement. Lesional and perilesional biopsies were obtained for hematoxylin and eosin stain (Fig 2) with direct immunofluorescence (DIF) negative for immunoproteins.

**Question 1: What is the best diagnosis?**

A. Bullous pemphigoid
B. Bullosis diabeticorum (BD)
C. Linear immunoglobulin A (IgA) bullous dermatosis
D. Bullous drug eruption
E. Bullous lupus erythematosus

**Answers:**

A. Bullous pemphigoid – Incorrect. Bullous pemphigoid presentation ranges between tense bullae to generalized itching or urticaria. Histopathology demonstrates eosinophilic subepidermal separation and DIF exhibits linear IgG and C3 along basement membrane.^1^
B. Bullosis diabeticorum (BD) – Correct. BD affects patients with poorly controlled diabetes presenting “overnight” as asymptomatic, tense bullae. Additionally, the irregular outlines of the bullae would be highly atypical for an immunobullous disease. Histopathologic findings in BD are nonspecific including intraepidermal or subepidermal blisters with minimal inflammation. DIF is negative and used to rule out other causes. This is an atypical presentation of BD in a pediatric patient involving the trunk in a linear distribution, as BD is typically seen affecting distal extremities in adults. Despite atypical location, no clinical differences, including healing of bullae, were noted in this patient.^2^^,^^3^
C. Linear IgA bullous dermatosis – Incorrect. Linear IgA bullous dermatosis presents with annular, polycyclic or herpetiform tense vesicles or bullae, and urticarial plaques. Histopathology demonstrates neutrophil-rich subepidermal separation and DIF exhibits linear IgA along basement membrane zone.^4^
D. Bullous drug eruption – Incorrect. Insulin is not a common culprit of bullous drug eruption.
E. Bullous lupus erythematosus – Incorrect. Bullous lupus erythematosus is seen in patient with history of systemic lupus. Histopathology demonstrates neutrophil-predominant subepidermal separation and DIF is positive for multiple immunoreactants (IgG, C3, IgA, IgM) in a granular pattern along the basement membrane.^5^

**Question 2: What is a first-line treatment option for this disease process?**

A. High-dose prednisone
B. Dapsone
C. Aspirate the fluid and apply mupirocin ointment
D. Oral doxycycline
E. Intravenous immunoglobulin

**Answers:**

A. High-dose prednisone – Incorrect. BD is not an inflammatory process. Steroids would worsen hyperglycemia and increase risk of infection.
B. Dapsone – Incorrect. Although in this patient biopsies rare neutrophils were present, neutrophils are not thought to be the primary driving force of the disease process as biopsies typically show minimal to no inflammation.
C. Aspirate the fluid and apply mupirocin ointment – Correct. Although treatment is not required as it is a self-limiting disease, some use a small-bore needle to aspirate fluid and apply topical antibiotics to prevent secondary infections while others leave the bullae intact.^2^
D. Oral doxycycline – Incorrect. BD is not an inflammatory or infectious process. Oral antibiotics would only be required if secondary infection was suspected.^2^
E. Intravenous immunoglobulin – Incorrect. BD is not an autoantibody mediated disease.

**Question 3: What is the typical sequalae of this disease process?**

A. Resolution of bullae with localized scarring
B. Diffuse spreading of bullae and vesicles
C. Develop systemic symptoms including fever and malaise
D. Nonscarring, relapsing, and remitting bullae
E. Chronic bullae formation

**Answers:**

A. Resolution of bullae with localized scarring – Incorrect. While the level of separation of the skin in BD can occur at different levels, the dermis remains intact and therefore scarring does not occur.^2^^,^^3^
B. Diffuse spreading of bullae and vesicles – Incorrect. While the etiology of BD remains unclear, it is uncommon for bullae to spread diffusely.^2^
C. Develop systemic symptoms including fever and malaise – Incorrect. BD is not associated with systemic symptoms. If present though, it can be a sign of an underlying infection as diabetic patients are at increased risk of infection.^2^
D. Nonscarring, relapsing, and remitting bullae – Correct. No scarring develops unless repeated episodes with ulceration occurs. The bullae can reappear at the same location or different locations with future episodes.^2^^,^^3^
E. Chronic bullae formation – Incorrect. The bullae self-resolve over weeks without treatment. Typically, once an episode resolves, there is a period of remission before additional bullae form.^3^

## Conflict of interest

None disclosed.
